# gMCSpy: efficient and accurate computation of genetic minimal cut sets in Python

**DOI:** 10.1093/bioinformatics/btae318

**Published:** 2024-05-15

**Authors:** Carlos J Rodriguez-Flores, Naroa Barrena, Danel Olaverri-Mendizabal, Idoia Ochoa, Luis V Valcárcel, Francisco J Planes

**Affiliations:** Tecnun School of Engineering, Biomedical Engineering and Sciences Department, University of Navarra, San Sebastián 20018, Spain; Tecnun School of Engineering, Biomedical Engineering and Sciences Department, University of Navarra, San Sebastián 20018, Spain; Tecnun School of Engineering, Biomedical Engineering and Sciences Department, University of Navarra, San Sebastián 20018, Spain; Tecnun School of Engineering, Biomedical Engineering and Sciences Department, University of Navarra, San Sebastián 20018, Spain; Biomedical Engineering Center, University of Navarra, Pamplona, Navarra 31009, Spain; Instituto de Ciencia de los Datos e Inteligencia Artificial (DATAI), University of Navarra, Pamplona 31080, Spain; Tecnun School of Engineering, Biomedical Engineering and Sciences Department, University of Navarra, San Sebastián 20018, Spain; Biomedical Engineering Center, University of Navarra, Pamplona, Navarra 31009, Spain; Instituto de Ciencia de los Datos e Inteligencia Artificial (DATAI), University of Navarra, Pamplona 31080, Spain; Tecnun School of Engineering, Biomedical Engineering and Sciences Department, University of Navarra, San Sebastián 20018, Spain; Biomedical Engineering Center, University of Navarra, Pamplona, Navarra 31009, Spain; Instituto de Ciencia de los Datos e Inteligencia Artificial (DATAI), University of Navarra, Pamplona 31080, Spain

## Abstract

**Motivation:**

The identification of minimal genetic interventions that modulate metabolic processes constitutes one of the most relevant applications of genome-scale metabolic models (GEMs). The concept of Minimal Cut Sets (MCSs) and its extension at the gene level, genetic Minimal Cut Sets (gMCSs), have attracted increasing interest in the field of Systems Biology to address this task. Different computational tools have been developed to calculate MCSs and gMCSs using both commercial and open-source software.

**Results:**

Here, we present gMCSpy, an efficient Python package to calculate gMCSs in GEMs using both commercial and non-commercial optimization solvers. We show that gMCSpy substantially overperforms our previous computational tool GMCS, which exclusively relied on commercial software. Moreover, we compared gMCSpy with recently published competing algorithms in the literature, finding significant improvements in both accuracy and computation time. All these advances make gMCSpy an attractive tool for researchers in the field of Systems Biology for different applications in health and biotechnology.

**Availability and implementation:**

The Python package gMCSpy and the data underlying this manuscript can be accessed at: https://github.com/PlanesLab/gMCSpy.

## 1 Introduction

The large number of interrelated reactions that support life makes the characterization of biological systems a daunting task. Genome-scale metabolic models (GEMs) have emerged in the last two decades to address this complexity. In particular, GEMs provide a comprehensive representation of the metabolic and genetic interplay in an organism, aiming to offer a holistic view of cellular metabolism by integrating genomic, biochemical, and physiological information (Gu *et al.* 2019). Importantly, different molecular layers in GEMs are connected via gene-protein-reaction (GPRs) rules, which describe how genes translate into the enzymes of specific reactions that produce/consume metabolites. In recent years, the field of systems biology has witnessed significant advancements in the analysis of GEMs, with a particular focus on identifying potential intervention strategies for different clinical and biotechnological applications.

An influential concept for the identification of optimal intervention strategies in GEMs is Minimal Cut Sets (MCSs). MCSs define a minimal (irreducible) set of reactions whose deletion leads to a desired metabolic phenotype, e.g. infeasible biomass production or optimal production of a compound of biotechnological interest (Klamt and Gilles 2004). We introduced a closely related concept called genetic Minimal Cut Sets (gMCSs), which define minimal intervention strategies at the gene level (Apaolaza *et al.* 2017). Different algorithms have been developed to calculate both MCSs and gMCSs in large GEMs (von Kamp and Klamt 2014, Pratapa *et al.* 2015, Schneider *et al.* 2020). In particular, we developed a function in the COBRA toolbox (Heirendt *et al.* 2019) to carry out this task (Apaolaza *et al.* 2019), called here GMCS. More recently, StrainDesign was released (Schneider *et al.* 2022), a Python library that improves previous developments of the same group and extends their framework to an open-source platform.

Here, we present gMCSpy, a novel Python package that calculates gMCSs for GEMs. gMCSpy integrates several algorithmic improvements with respect to our previous tool, GMCS, which was built in MATLAB environment. Furthermore, gMCSpy allows the user to search for gMCSs with both commercial and open-source Mixed Integer Linear Programming (MILP) (optimization) solvers. We show that gMCSpy overperforms GMCS and StrainDesign in computation time and scalability in a benchmark of seven relevant GEMs. Overall, in our attempt to release an open-source framework, gMCSpy demonstrates computational and accuracy advances with respect to previous methods in the literature.

## 2 Materials and methods

gMCSpy is an open-source package written in Python to calculate gMCSs in GEMs. The package was built using COBRApy (Ebrahim *et al.* 2013) to conform with the standardization of GEMs and take advantage of model manipulations previously developed by the COBRA community.

In order to compute gMCSs, we implemented the Mixed Integer Linear Programming (MILP) model defined in the work of Apaolaza *et al.* (2019) (Supplementary Methods). gMCSpy includes a common (developed in-house) interface to define this MILP model and translation functions to compute gMCSs with different solvers, namely the commercial solvers Gurobi (Gurobi Optimization LLC 2023) and IBM ILog CPLEX (Cplex II 2009), and the open-source solver SCIP (Bestuzheva *et al.* 2023). This constitutes an advance with respect to our previous tool, GMCS, which was developed in MATLAB environment exclusively for CPLEX. Moreover, gMCSpy can use the latest versions of CPLEX, currently V22.1.0, in contrast, the latest version of CPLEX compatible with MATLAB is V12.10.0.

A critical part in our methodology is the computation of matrix G, which defines the gene knockout constraints in our MILP model (see Supplementary Methods). This step is performed by the function buildGMatrix, which has been substantially improved in gMCSpy with respect to our previous work (Apaolaza *et al.* 2019). In particular, buildGMatrix requires the function parseGPRToModel, which transforms GPR rules into artificial reaction networks (GPR networks) (Apaolaza *et al.* 2019, Barrena *et al.* 2023). We have implemented an efficient recursive strategy in Python to reduce the computational expenditure in constructing GPR networks. This function allows us to efficiently deal with the most complex GPR rules in published GEMs (see Table 1), in contrast to other methods that limit the size of GPR rules that they can handle (Schneider *et al.* 2022).

**Table 1.** Summary of GEMs used in the benchmarking study of gMCS computation.

Model	No. reactions	No. metabolites	No. genes	Largest GPR (Gene set)	Largest GPR (Number of Disjunctions: And-Or)
*E.coli* core	95	72	137	13	16
iML1515	2712	1877	1516	17	17
iJN1463	2927	2153	1462	14	23
Yeast-GEM-8.7	4131	2806	1163	20	57
Human-GEM-1.16	11 944	7118	2897	58	231
Human-GEM-1.16.-Media	11 509	6800	2897	58	231
Recon3D	10 600	5835	2248	53	5162^a^

Dimension of each considered GEM in terms of number of reactions, metabolites, and genes. The last two columns detail the largest GPR rule for each GEM, considering the number of genes and the number of disjunctions (And-Or).

a Note that Recon3D uses pseudo-transcripts instead of genes, and GPR rules must be transformed into gene domain. For this reason, GPR rules can be abnormally longer.

The main function to calculate gMCSs in gMCSpy is calculateGeneMCS. Importantly, the search strategy was refined to more accurately enumerate gMCSs in increasing length order (Supplementary Methods). This function can be used to perform a global search (with all genes) but also to identify gMCSs involving a specific subset of genes, as done in Apaolaza *et al.* (2017), with the targetKOs attribute. Likewise, we can compute nutrient-genetic MCSs (ngMCSs), interventions that combine nutrient deprivations in the environment and gene knockouts (Apaolaza *et al.* 2022), using the isNutrient attribute. The same analysis can be done at the reaction level with the function calculateMCS. Different examples are available in the gMCSpy documentation to illustrate these functions (https://planeslab.github.io/gMCSpy/).

### 2.1 Benchmarking study 

The performance of gMCSpy (V1.0.0) was benchmarked with two published tools in the literature: our previous MATLAB tool, GMCS (*calculateGeneMCS* function of the COBRA Toolbox V3.4), and StrainDesign (V1.11). gMCSpy and StrainDesign were compared with two commercial solvers: CPLEX (V22.1.0) and Gurobi (V11.0); while GMCS was examined only with CPLEX (V12.10.0), as it is not designed to work with Gurobi. The open-source solver SCIP (V8.0.0) was assessed individually with gMCSpy. Specifically, for the different considered approaches, we compared their capacity of extracting all single, double, and triple gene deletions which result to be lethal for proliferation, as well as the required computation time in 10 runs, for 7 GEMs of different size. All experiments were performed in an Intel Xeon Gold 6248R processor using 16 threads and 64 GB of RAM.

The following GEMs were considered in our benchmarking study: *E.coli* core (Orth *et al.* 2010a) and the most recent GEMs of *E.coli*, iML1515 (Monk *et al.* 2017); *Pseudomonas putida*, iJN1463 (Nogales *et al.* 2020); *S.cerevisiae*, Yeast-GEM v8.7.0 (Lu *et al.* 2019); and human cells, Recon3D (Brunk et al., 2018) and Human-GEM-1.16 (Robinson *et al.* 2020). In the case of Human-GEM, we considered two cases: under the most general growth medium (Human-GEM-1.16) and under Ham’s growth medium (Human-GEM-1.16-Media). Using the RAVEN toolbox, we applied the Ham’s growth medium and removed all reactions and associated metabolites that cannot undertake any flux in this setting (Wang *et al.* 2018). Full details can be found in Table 1.

Finally, in order to systematically quantify the impact in computation time of different approaches and solvers, we conducted one-sided paired Wilcoxon tests (Wilcoxon 1945), detailing *P*-values and estimated log2(fold-change).

## 3 Results

**Figure 1.**
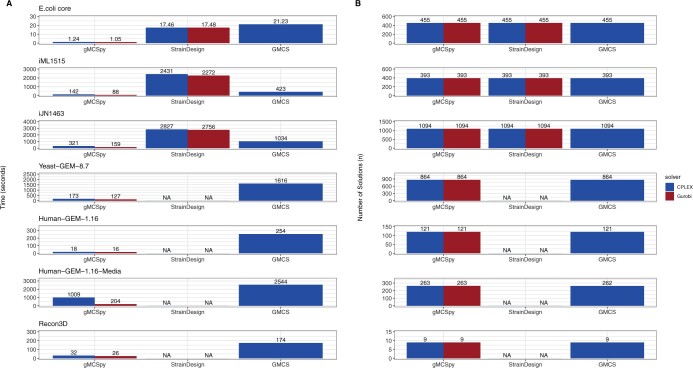
Benchmark of existing tools to compute gMCS in GEMs. (A) Computation times to calculate gMCS up to length 3, i.e. lethal single, double, and triple knockouts, for each of the cases analyzed with gMCSpy, StrainDesign, and GMCS. Mean values across 10 different runs are shown at the top of bars. “NA” (not applicable) means that the calculation was not possible. The standard deviation across runs is shown in Supplementary Table S1; (B) Number of gMCSs up to length 3 that were found for each of the cases analyzed with gMCSpy, StrainDesign, and GMCS.

The results obtained from the benchmarking study described above are summarized in Fig. 1. Note here that we found that SCIP takes considerably longer than CPLEX and Gurobi in the GEMs considered (*P*-value = 9.5e-7, log2(fold-change) = 9.66). For instance, gMCSpy with SCIP took 5571 s on average to solve the *E.coli* core model, while only 1 s with commercial solvers. To simplify the discussion, the performance of gMCSpy with SCIP is shown separately in Supplementary Fig. S1, where it can be observed that SCIP is still far from commercial solvers. The main conclusions for the rest of approaches with CPLEX and Gurobi are presented below.

First, we note that not all GEMs were compatible with StrainDesign (Table 1). This incompatibility comes from GPR rules, as StrainDesign can only handle GPR rules with a limited complexity. This implies that complex GPR rules need to be removed before using StrainDesign, in contrast to gMCSpy or GMCS, which can efficiently deal with the most complex GPR rules found in the GEMs under study, e.g. a GPR rule in Recon3D involving 53 genes and 5162 disjunctions (Table 1).

Second, as shown in Fig. 1A, gMCSpy substantially reduces the computation time with respect to StrainDesign in all the GEMs tested (*P*-value = 0.015, log2(fold-change) = −4). In some of the scenarios, we obtained improvements of nearly 20-fold increase in speed with respect to StrainDesign, e.g. log2(fold-change) = −4.68 with Gurobi in iML1515. Moreover, gMCSpy obtained a better performance in Gurobi than in CPLEX in most cases (*P*-value = 0.007, log2(fold-change) = −0.74). This is not the case in StrainDesign, where both solvers had a similar behavior (*P*-value = 0.25, log2(fold-change) = 0.04).

With respect to our previous tool, GMCS, we observed several performance improvements. First, as mentioned in Section 2, a key improvement of gMCSpy over GMCS is related to the computation of matrix G. We found significant differences in computation time between GMCS and gMCSpy in the calculation of matrix G (*P*-value = 0.007, log2(fold-change) = −3.81) (Supplementary Fig. S2). For example, gMCSpy took on average 3.18 s to build G matrix in Human-GEM-1.16-Media while GMCS took 93.24 s.

Second, we slightly modified the search process of gMCSs to avoid the case found in Human-GEM-1.16-Media, where one lethal triple gene knockout was missed with GMCS (Fig. 1B) (see Supplementary Methods). This modification in our approach has a negligible impact on the computation performance of gMCSpy (*P*-value = 0.23, log2(fold-change) = 0.12, Supplementary Fig. S3). Note here that, in order to further substantiate that gMCSpy can calculate all gMCSs, we conducted an exhaustive brute force screening of all possible single and double gene knockout combinations and assessed biomass production with Flux Balance Analysis (Orth *et al.* 2010b), finding the same lethal combinations as gMCSpy. The analysis of lethal triple gene knockout combinations was discarded due to the high computational expense for all GEMs aside from *E.coli* core.

Third, gMCSpy shows a superior performance across all models with respect to GMCS (*P*-value = 0.007, log2(fold-change) = −2.59), presenting more than 10-fold reduction in computation time in some scenarios tested (e.g. log2(fold-change) = −3.66 with Yeast-GEM-8.7 in Gurobi). These results clearly show that gMCSpy is more efficient and accurate in computing gMCSs than our previous MATLAB tool, GMCS.

## 4 Discussion

The capacity of predicting key genetic interventions has profound implications for various areas in health and biotechnology. The gMCS framework offers a valuable tool for optimizing interventions within biological systems, particularly in the field of drug discovery, where it can aid in the identification of potential drug targets that modulate cellular metabolism in different disease areas, opening new avenues for the development of personalized medicine.

gMCSpy constitutes an efficient open-source Python package to calculate gMCSs in GEMs using both commercial (CPLEX and Gurobi) and non-commercial (SCIP) optimization solvers. Despite the continuous improvement of non-commercial optimization solvers, the performance of gMCSpy with CPLEX and Gurobi is still far from SCIP, particularly for GEMs of high size and number of gMCSs. Using commercial solvers, we show that gMCSpy substantially improves our previous tool, GMCS, which was developed in MATLAB environment and limited to CPLEX, as shown in the benchmarking study summarized in Fig. 1. Moreover, gMCSpy overperforms in both accuracy and computation time StrainDesign, a competing algorithm in the literature. All these advances make gMCSpy an attractive tool for researchers in the field of Systems Biology.

## Supplementary Material

btae318_Supplementary_Data
