# Supplementary material for: gMCSpy: efficient and accurate computation of genetic minimal cut sets in Python
Source: Bioinformatics. 2024 May 15;40(6):btae318. doi: 10.1093/bioinformatics/btae318 (PMC11199197; doi:10.1093/bioinformatics/btae318)
Supplement: btae318_Supplementary_Data [file btae318_supplementary_data.pdf]

Supplementary Material: gMCSpy: Genetic Minimal Cut Sets in Python

Carlos Javier Rodriguez[1], Naroa Barrena[1], Danel Olaverri-Mendizabal[1], Idoia Ochoa[1,2,3], Luis V. Valcarcel[1,2,3] and Francisco J. Planes[1,2,3*]

[1]University of Navarra, Tecnun School of Engineering, Manuel de Lardizábal 13, 20018 San Sebastián, Spain.

[2]University of Navarra, Biomedical Engineering Center, Campus Universitario 31009 Pamplona, Navarra, Spain.

[3]University of Navarra, Instituto de Ciencia de los Datos e Inteligencia Artificial (DATAI), Campus Universitario, 31080, Pamplona, Spain

[*]*Corresponding authorj: fplanes@tecnun.es*

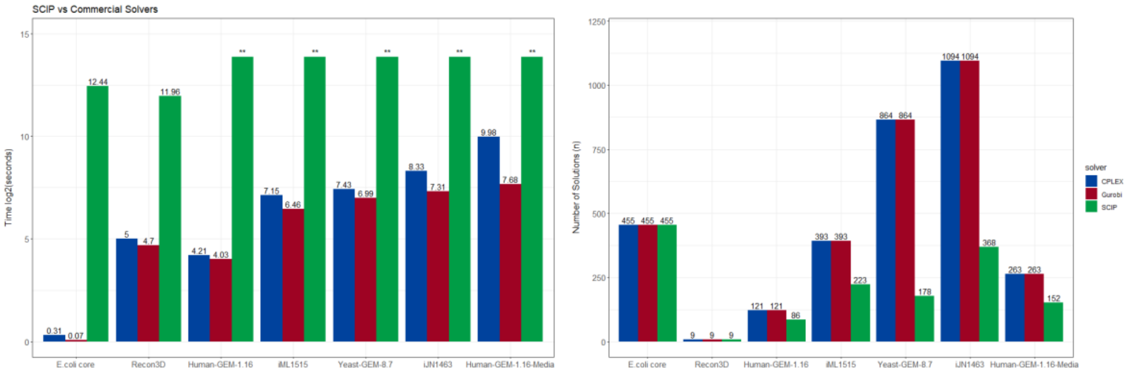

Supplementary Figure 1: SCIP performance with *gMCSpy*. We present the performance of *gMCSpy* using the optimization solver *SCIP* (Bestuzheva et al., 2023) for different GEMs considered in the main text: *E. coli* core (Orth, Fleming, et al., 2010) and the most recent GEMs of *E. coli*, iML1515 (Monk et al., 2017); *P. putida*, iJN1463 (Nogales et al., 2020); *S. cerevisiae*, Yeast-GEM v8.7.0 (Lu et al., 2019); and human cells, Recon3D (Brunk et al, 2018) and Human-GEM-1.16 (Robinson et al., 2020). In the case of Human-GEM, we considered two cases: under the most general growth medium (Human-GEM-1.16) and under Ham's growth medium (Human-GEM-1.16-Media). Results are shown for the computation of gMCS up to order 3. The difference in computation time with respect to Gurobi *and* CPLEX *is* very relevant: one-sided paired Wilcoxon test (Wilcoxon, 1945), p-value=9.5e-7 and log2(fold-change) of 9.65, only considering the cases for which SCIP obtained the full set of solutions. ** Cases where SCIP exceeded the time limit of 10,000 seconds. It can be observed that for these cases the full set of solutions was not obtained within this time window.

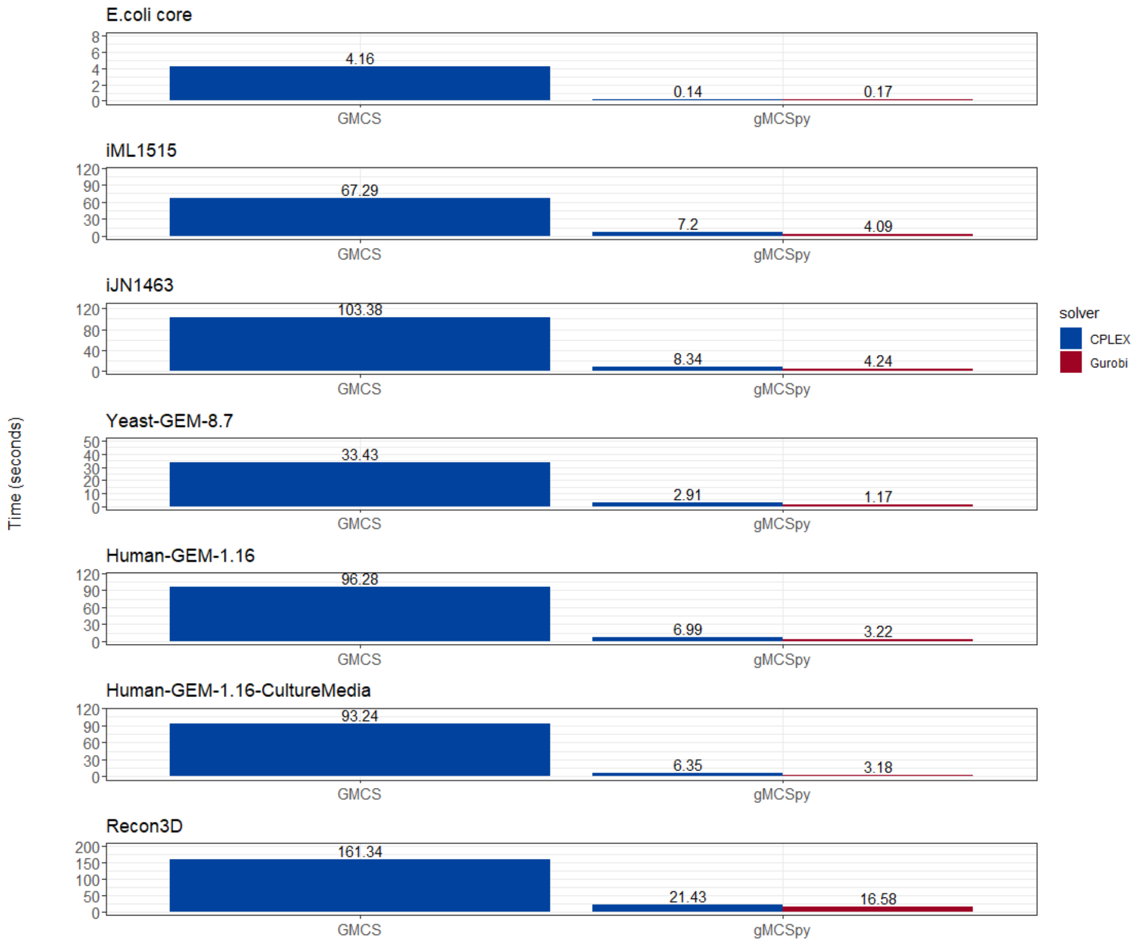

Supplementary Figure 2: Computation time for the calculation of matrix G with *gMCSpy* and *GMCS*. We considered in this study *E. coli core* (Orth et al., 2010) and the most recent GEMs of *E. coli*, *P. putida, S. cerevisiae* and human cells: iML1515 (Monk et al., 2017), iJN1463 (Nogales et al., 2020), Yeast-GEM v8.7.0 (Lu et al., 2019), Human-GEM v1.16.0 (Robinson et al., 2020) and Recon3D (Brunk et al., 2018). In the case of human cells, we considered two cases: under the most general growth medium (Human-GEM v1.16.0) and under Ham's growth medium (Human-GEM v1.16.0_CultureMedia). For each GEM, we calculated the G matrix with *gMCSpy* and *GMCS* and measured the execution time (in seconds) with the 2 commercial solvers: CPLEX and Gurobi.

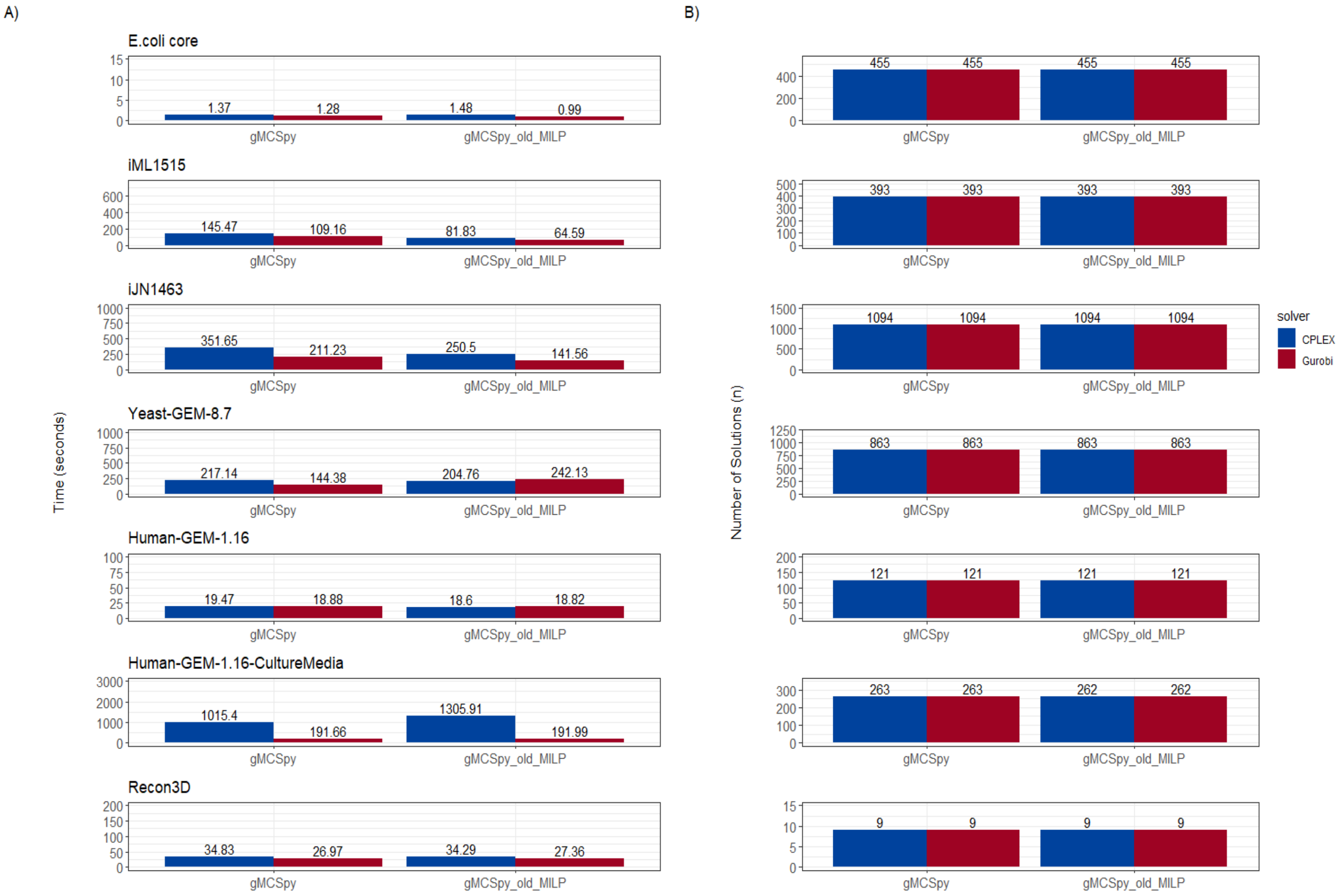

Supplementary Figure 3: Performance of *gMCSpy* with different methodologies. As noted in the main text, we implemented in *gMCSpy* the Mixed Integer Linear Programming (MILP) model defined in the work of Apaolaza et al., 2019, with a slight modification in the definition of matrix G that allows us to more accurately enumerate gMCSs in increasing length order. We compared here the performance of *gMCSpy* with our previous methodology (*gMCSpy_old_MILP*) and our new methodology (*gMCSpy*) with the two commercial solvers: CPLEX and Gurobi. Again, we considered *E. coli core* (Orth et al., 2010) and the most recent GEMs of *E. coli*, *P. putida, S. cerevisiae* and human cells: iML1515 (Monk et al., 2017), iJN1463 (Nogales et al., 2020), Yeast-GEM v8.7.0 (Lu et al., 2019), Human-GEM v1.16.0 (Robinson et al., 2020) and Recon3D (Brunk et al., 2018). In the case of human cells, we considered three cases: under the most general growth medium (Human-GEM v1.16), under Ham's growth medium (Human-GEM v1.16-CultureMedia). A) Computation times (in seconds) to calculate gMCS up to length 3. *i.e.* lethal single, double and triple knockouts, for each of the cases analyzed with *gMCSpy* and *gMCSpy_old_MILP*. Mean values across 10 different runs are shown at the top of bars. B) Number of gMCSs up to length 3 that were found for each of the cases analyzed with *gMCSpy* and *gMCSpy_old_MILP*.

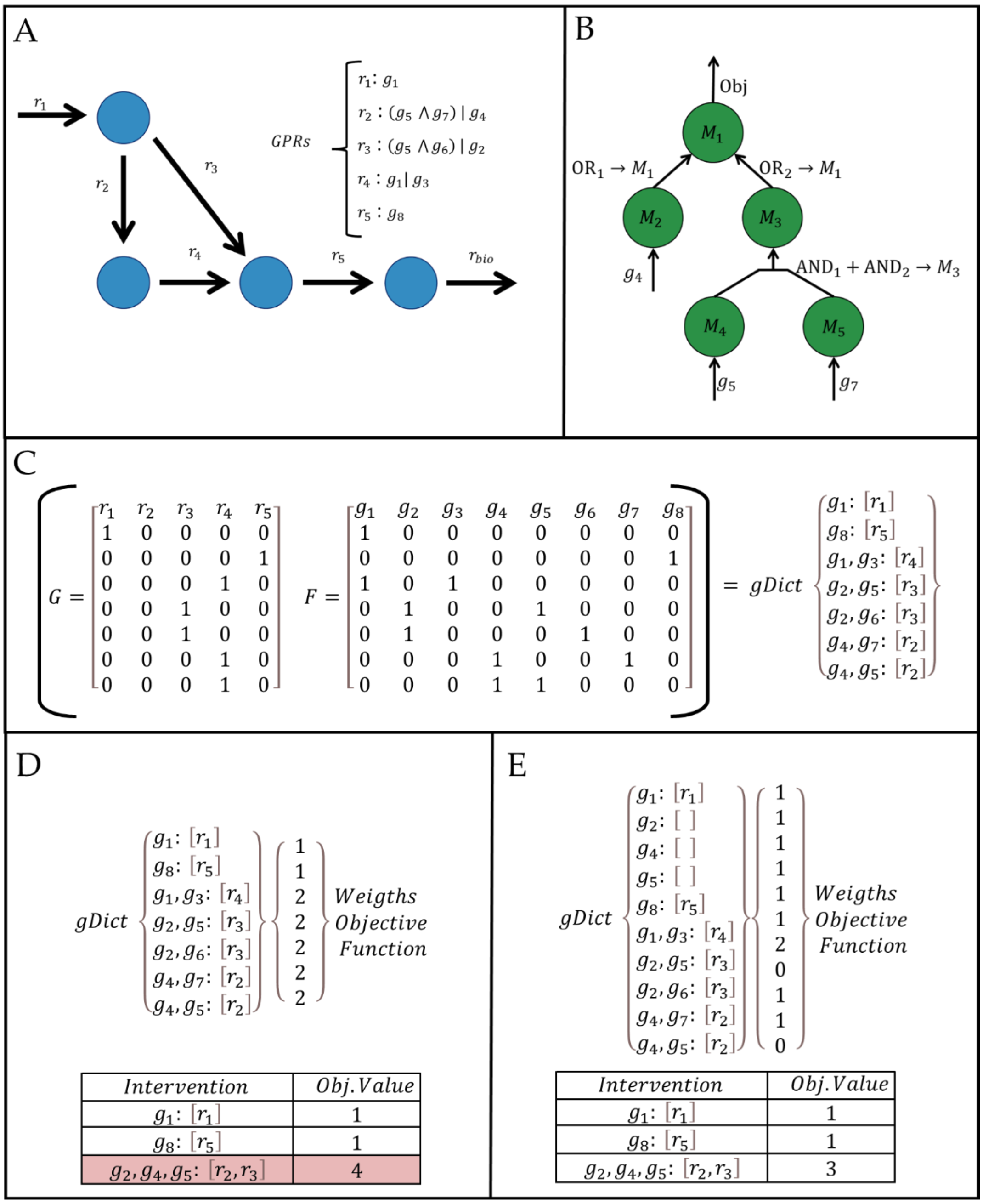

Supplementary Figure 4: Toy example to illustrate the calculation of G matrix. A) Example metabolic network involving 6 six reactions: r_1, r_2, r_3, r_4, r_5 and r_{bio}, and 8 genes: g_1, g_2, g_3, g_4, g_5, g_6, g_7 and g_8 in GPR rules; B) Example GPR network for r_2. GPR networks are built using an efficient recursive function using Boolean tress available in COBRApy; C) Resulting G and F matrices, as well as data structure exemplifying the equivalent GDict for our toy example. D) One example of the expansion process between interventions, if the intersection of two keys yields a non-existing key in the GDict it is added as a null row. E) The equivalent GDict in methodology presented in Apaolaza et al. 2019 and associated weights in the objective function. In the toy example, the intervention $\{g_2, g_4, g_5\}$ would NOT be discovered when computing all single, double and triple knockouts. E) The GDict generated in gMCSpy inserting intersecting genes mitigates this eventuality increasing the accuracy of our methodology.

Supplementary Table 1: Details of computation time for different approaches and commercial solvers. Mean time and standard deviation across 10 runs of each case benchmarked in Figure 1; number of solutions obtained for each case. NA (Not applicable) is used on the set of runs where the algorithms where not capable of handling the model.

Model	Solver	Approach	Mean Time (seconds)	Standard Deviation Time	Number of Solutions
E.coli core	CPLEX	GMCS	21.23	1.80	455
Human-GEM-1.16	CPLEX	GMCS	253.82	1.86	121
Human-GEM-1.16-Media	CPLEX	GMCS	2,544.41	1.41	262
Recon3D	CPLEX	GMCS	174.04	2.43	9
Yeast-GEM-8.7	CPLEX	GMCS	1,616.29	4.28	864
iJN1463	CPLEX	GMCS	1,034.36	2.16	1094
iML1515	CPLEX	GMCS	423.30	2.97	393
E.coli core	CPLEX	gMCSpy	1.24	0.19	455
E.coli core	Gurobi	gMCSpy	1.05	0.33	455
Human-GEM-1.16	CPLEX	gMCSpy	18.47	0.49	121
Human-GEM-1.16	Gurobi	gMCSpy	16.28	0.62	121
Human-GEM-1.16-Media	CPLEX	gMCSpy	1,008.84	2.03	263
Human-GEM-1.16-Media	Gurobi	gMCSpy	204.42	0.66	263
Recon3D	CPLEX	gMCSpy	31.94	0.73	9
Recon3D	Gurobi	gMCSpy	25.60	0.66	9
Yeast-GEM-8.7	CPLEX	gMCSpy	172.97	0.31	864
Yeast-GEM-8.7	Gurobi	gMCSpy	127.48	0.51	864
iJN1463	CPLEX	gMCSpy	321.43	1.32	1094
iJN1463	Gurobi	gMCSpy	158.79	0.96	1094
iML1515	CPLEX	gMCSpy	141.56	0.53	393
iML1515	Gurobi	gMCSpy	88.03	0.29	393
E.coli core	CPLEX	StrainDesign	17.46	0.79	455
E.coli core	Gurobi	StrainDesign	17.48	0.43	455
Human-GEM-1.16	CPLEX	StrainDesign	NA	NA	NA
Human-GEM-1.16	Gurobi	StrainDesign	NA	NA	NA
Human-GEM-1.16-Media	CPLEX	StrainDesign	NA	NA	NA
Human-GEM-1.16-Media	Gurobi	StrainDesign	NA	NA	NA
Recon3D	CPLEX	StrainDesign	NA	NA	NA
Recon3D	Gurobi	StrainDesign	NA	NA	NA
Yeast-GEM-8.7	CPLEX	StrainDesign	NA	NA	NA
Yeast-GEM-8.7	Gurobi	StrainDesign	NA	NA	NA
iJN1463	CPLEX	StrainDesign	2,827.42	105.65	1094
iJN1463	Gurobi	StrainDesign	2,755.79	33.80	1094
iML1515	CPLEX	StrainDesign	2,430.71	28.21	393
iML1515	Gurobi	StrainDesign	2,271.62	96.34	393

Supplementary Methods

MILP formulation for the calculation of gMCSs

In this section, we present a brief description of the mixed integer linear programming (MILP) model, previously developed in different works of our group (Apaolaza et al., 2017, 2019; Barrena et al., 2023), which allows us to search for genetic minimal cut sets (gMCS) in genome-scale metabolic models (GEMs).

For a particular GEM, the full set of reactions is typically represented with the stoichiometric matrix, here denoted as S, of dimensions $m \times n$, where m and n are the total number of metabolites and reactions, respectively. The values of each column represent the stoichiometric coefficients of the different metabolites in a particular reaction. Products and substrates in a reaction take positive and negative coefficients, respectively. Each reaction can carry a flux r; however, we do no not allow negative fluxes and, thus, each reversible reaction must be split into two irreversible reactions:

$$r \geq 0 \quad \text{(Eq.1)}$$

Considering the steady state and the mass balance equation, we can establish that consumption and production fluxes must be equal to zero:

$$S \cdot r = 0 \quad \text{(Eq.2)}$$

Next, we define the target metabolic task that we aim to block, typically the biomass reaction. Thus, we force a positive flux through this target task:

$$t^T \cdot r \geq t^* \quad \text{(Eq.3)}$$

where t^Trepresents a row vector in which all its elements are zero except for the reactions implied in the task; and t^* is a positive constant.

Eq. 4 defines the gene knockout constraints. As detailed in the next section, each row in G specifies the subset of reactions blocked as a result of a minimal subset of gene knockouts, stored in *F* matrix.

$$G \cdot r \ \leq 0 \quad (Eq.4)$$

Eqs. 1-4 define the primal linear programming problem. Due to the inconsistency between Eq.3 and Eq.4, the primal problem is infeasible. In that situation, its associated dual problem defines an unbounded polyhedral cone, whose extreme rays represent minimal subsets of constraints in the primal problem that are inconsistent and lead to the underlying infeasibility. This is closely related with the concept of gMCSs, which are defined by Eq.3 and minimal subsets of gene knockout constraints in Eq.4. Accordingly, gMCSs can be calculated using the following MILP:

$$minimize \sum_{i=1}^{i=l} d_i \cdot z_i \quad \text{(Eq.5)}$$

$$s.t.$$

$$N \cdot \begin{pmatrix} u \\ v \\ w \end{pmatrix} = [S^T \ G^T - t] \cdot \begin{pmatrix} u \\ v \\ w \end{pmatrix} \geq 0 \quad \text{(Eq.6)}$$

$$\alpha \cdot z \leq v \leq M \cdot z \qquad \text{(Eq.7)}$$

$$r^* \cdot w \leq -c, c > 0 \quad \text{(Eq.8)}$$

$$z_\delta \geq z_\beta \qquad \forall(\delta, \beta) |\, F(\beta) \supset F(\delta) \quad \text{(Eq.9)}$$

$$\sum_{i=1}^{i=l} z_i^j z_i \leq \sum_{i=1}^{i=l} z_i^j - 1 \quad \text{(Eq.10)}$$

$$v \geq 0; w \geq 0 \quad \text{(Eq.11)}$$

$$u \in R^m, v \in \ R^l, w \in R, z \ \in B^l \quad \text{(Eq.12)}$$

where u, v, and w denote dual variables linked to the mass balance equation, gene knockout constraints, and the target (metabolic task) constraint, respectively. Eq. 6 defines the constraints associated to the dual problem. In addition, z variables are binary variables associated with v variables through Eq.7, which guarantees that if $z = 1$ then $v > \alpha$ or if $z = 0$ then $v = 0$. Eq. 8 forces w to be non-zero, incorporating the target constraint, Eq.3, into the infeasible primal problem. Eq.9 addresses the dependencies between dual variables v, which may yield non-

minimal solutions. Finally, Eq.10 eliminates previous solutions (z^j) from the solution space and enables the enumeration of gMCSs in increasing number of gene knockout. Eqs. 11-12 define the nature of variables used in our MILP. The objective function, Eq.5, is discussed below.

Calculation of matrix G

In practice, before formulating the MILP problem, we need to define matrix G, which is required for the construction of the gene knockout constraints in Eq. 4. Each row in the G matrix is linked to a minimal subset of gene knockouts that disables a specific subgroup of reactions. In *gMCSpy*, for the definition of matrix G, we use a Python dictionary, data structure composed by key-value pairs, where each key must be unique and associated with a specific value. In our implementation, keys represent a subset of genes and values a list of reactions. We call the resulting Python dictionary *GDict*.

For illustration, consider the example network depicted in Supplementary Figure 4A, which involves 6 reactions, 4 metabolites and 8 genes in GPR rules. For the construction of *GDict*, we first focus on reactions controlled only by one gene, in our case r_1 and r_5. Next, we consider reactions that are only controlled by AND relationships followed by reactions with only OR relationships (only r_4 in the toy example). Finally, we deal with more complex GPRs, such as r_2 and r_3. For it, we first convert GPRs into Boolean trees using *COBRApy* (Ebrahim et al., 2013). The, we apply an efficient recursive function (developed in *gMCSpy*) that transforms Boolean trees into artificial reaction networks (GPR networks), as shown in Supplementary Figure 4B. We then search for MCSs that disrupts the target reaction in these GPR networks using the method described in Apaolaza et al., 2019, based on Minimal Cut Sets.

Calculation of G matrix

The resulting *GDict* in our toy example can be observed in Supplementary Figure 4C. *GDict* condenses all the information required to build Eq.4 and Eq.6 in our MILP into a single data structure, in contrast with our previous MATLAB approach requiring the calculation of matrices G and F, also shown in Supplementary Figure 4C. Note here that each row in G defines a specific subset of reactions blocks for a minimal subset of gene knockouts, which are defined in its associated row in F.

Preprocessing expansion of interventions

The objective function in our MILP, Eq.5, aims to minimize the number of gene knockouts to block the target metabolic task, according to a weighting system of z variables that considers their dependencies, summarized in Eq.9. For illustration, in our toy example, the intervention associated with the first row in G and F, $\{g_1: [r_1]\}$, is contained in the intervention associated with the sixth row in G and F, $\{g_1, g_3: [r_4]\}$. Thus, we need to implement the following constraint: $z_1 - z_6 \geq 0$, which means that if we activate the intervention of the sixth row, $z_6 = 1$ (knockout of g_1 and g_3), then the intervention of the first row must be also active, $z_1 = 1$ (knockout of g_1).

As detailed in Apaolazaet al., 2019, these dependency constraints generate an imbalance in the objective function that requires adjusting the weights of each binary variable z_i in order to return exactly the number of genes involved in the output gMCS. In our previous methodology (GMCS), the weight of each variable z_i is defined as the number of genes involved in its associated intervention minus the number of genes involved in other dependent interventions of smaller length. For example, the weight of dual variable z_6 in the objective function is $d_6 = 2 - 1 = 1$, since we need to subtract the number of genes in its dependent intervention, z_1. The resulting weights for our toy example are shown in Supplementary Figure 4D.

The caveat of this methodology is that we may find situations where the objective does not coincide exactly with the number of gene knockouts. This explains why one of the gMCSs of length 3 in the case of *Human-GEM v1.16.0_CultureMedia* is missed, as summarized in Figure 1B in the main text. Supplementary Figure 4D also shows one of the resulting gMCSs, $\{g_2,g_4,g_5\}$, which incorrectly has an objective value of 4 and should be 3.

To address this issue, *gMCSpy* uses a different approach. In particular, we build a slightly modified G matrix and, therefore, *GDict*, as shown in Supplementary Figure 4E for our toy example. In particular, a null row is added to G for every gene that takes part in F matrix in more than one row but not as a single gene. This is the case in our toy example of the following genes: g_2, g_4 and g_5. For example, in the case of g_5, it participates in two rows of F, $\{g_2, g_5\}$ and $\{g_4, g_5\}$, but none of them as a single. This strategy requires a slightly larger G matrix and, thus, a higher number of dual variables. However, it corrects the weights of dual variables (Supplementary Figure 4E), guaranteeing that all solutions are recovered with the exact length of gene knockouts with a similar computational cost (Supplementary Figure 4E).

References

Apaolaza, I., San José-Eneriz, E., Tobalina, L., Miranda, E., Garate, L., Agirre, X., Prósper, F., & Planes, F. J. (2017). An in-silico approach to predict and exploit synthetic lethality in cancer metabolism. *Nature Communications 2017 8:1*, *8*(1), 1–9. https://doi.org/10.1038/s41467-017-00555-y

Apaolaza, I., Valcarcel, L. V., & Planes, F. J. (2019). GMCS: Fast computation of genetic minimal cut sets in large networks. *Bioinformatics*, *35*(3), 535–537. https://doi.org/10.1093/bioinformatics/bty656

Barrena, N., Valcárcel, L. V, Olaverri-Mendizabal, D., Apaolaza, I., & Planes, F. J. (2023). Synthetic lethality in large-scale integrated metabolic and regulatory network models of human cells. *Npj Systems Biology and Applications*, *9*(1), 32. https://doi.org/10.1038/s41540-023-00296-3

Bestuzheva, K., Besançon, M., Chen, W.-K., Chmiela, A., Donkiewicz, T., van Doornmalen, J., Eifler, L., Gaul, O., Gamrath, G., Gleixner, A., Gottwald, L., Graczyk, C., Halbig, K., Hoen, A., Hojny, C., van der Hulst, R., Koch, T., Lübbecke, M., Maher, S. J., … Witzig, J. (2023). Enabling Research through the SCIP Optimization Suite 8.0. *ACM Trans. Math. Softw.*, *49*(2). https://doi.org/10.1145/3585516

Brunk, E., Sahoo, S., Zielinski, D. C., Altunkaya, A., Dräger, A., Mih, N., Gatto, F., Nilsson, A., Preciat Gonzalez, G. A., Aurich, M. K., Prlic, A., Sastry, A., Danielsdottir, A. D., Heinken, A., Noronha, A., Rose, P. W., Burley, S. K., Fleming, R. M. T., Nielsen, J., … Palsson, B. O. (2018). Recon3D enables a three-dimensional view of gene variation in human metabolism. *Nature Biotechnology 2018 36:3*, *36*(3), 272–281. https://doi.org/10.1038/nbt.4072

Ebrahim, A., Lerman, J. A., Palsson, B. O., & Hyduke, D. R. (2013). COBRApy: COnstraints-Based Reconstruction and Analysis for Python. *BMC Systems Biology*, *7*(1), 1–6. https://doi.org/10.1186/1752-0509-7-74/FIGURES/2

Lu, H., Li, F., Sánchez, B. J., Zhu, Z., Li, G., Domenzain, I., Marcišauskas, S., Anton, P. M., Lappa, D., Lieven, C., Beber, M. E., Sonnenschein, N., Kerkhoven, E. J., & Nielsen, J. (2019). A consensus S. cerevisiae metabolic model Yeast8 and its ecosystem for comprehensively probing cellular metabolism. *Nature Communications*, *10*(1). https://doi.org/10.1038/s41467-019-11581-3

Monk, J. M., Lloyd, C. J., Brunk, E., Mih, N., Sastry, A., King, Z., Takeuchi, R., Nomura, W., Zhang, Z., Mori, H., Feist, A. M., & Palsson, B. O. (2017). iML1515, a knowledgebase that computes Escherichia coli traits. *Nature Biotechnology 2017 35:10*, *35*(10), 904–908. https://doi.org/10.1038/nbt.3956

Nogales, J., Mueller, J., Gudmundsson, S., Canalejo, F. J., Duque, E., Monk, J., Feist, A. M., Ramos, J. L., Niu, W., & Palsson, B. O. (2020). High-quality genome-scale metabolic modelling of Pseudomonas putida highlights its broad metabolic capabilities. *Environmental Microbiology*, *22*(1), 255–269. https://doi.org/10.1111/1462-2920.14843

Orth, J. D., Fleming, R. M. T., & Palsson, B. Ø. (2010). Reconstruction and Use of Microbial Metabolic Networks: the Core Escherichia coli Metabolic Model as an Educational Guide . *EcoSal Plus*, *4*(1). https://doi.org/10.1128/ECOSALPLUS.10.2.1/FORMAT/EPUB

Robinson, J. L., Kocabaş, P., Wang, H., Cholley, P.-E., Cook, D., Nilsson, A., Anton, M., Ferreira, R., Domenzain, I., Billa, V., Limeta, A., Hedin, A., Gustafsson, J., Kerkhoven, E. J., Svensson, L. T., Palsson, B. O., Mardinoglu, A., Hansson, L., Uhlén, M., & Nielsen, J. (2020). An atlas of human metabolism. In *Sci. Signal* (Vol. 13). https://www.science.org

Wilcoxon, F. (1945). Individual Comparisons by Ranking Methods. *Biometrics Bulletin*, *1*(6), 80. https://doi.org/10.2307/3001968
